# Single-cell RNA sequencing data analysis suggests the cell–cell interaction patterns of the pituitary–kidney axis

**DOI:** 10.1038/s41598-022-14680-2

**Published:** 2022-07-01

**Authors:** Yiyao Deng, Jingjing Da, Jiali Yu, Chaomin Zhou, Jing Yuan, Yan Zha

**Affiliations:** 1 Department of Nephrology, Guizhou Provincial People’s Hospital, 83, Zhongshan Road, Nanming District, Guiyang, 550002 Guizhou China; 2 School of Medicine, Guizhou University, Guiyang, 550025 Guizhou China; 3 NHC Key Laboratory of Pulmonary Immunological Disease, Guizhou Provincial People’s Hospital, Guiyang, 550002 Guizhou China

**Keywords:** Cell biology, Physiology, Nephrology

## Abstract

Kidney functions, including electrolyte and water reabsorption and secretion, could be influenced by circulating hormones. The pituitary gland produces a variety of hormones and cytokines; however, the influence of these factors on the kidney has not been well explained and explored. To provide more in-depth information and insights to support the pituitary–kidney axis connection, we used mouse pituitary and kidney single-cell transcriptomics data from the GEO database for further analysis. Based on a ligand–receptor pair analysis, cell–cell interaction patterns between the pituitary and kidney cell types were described. Key ligand–receptor pairs, such as GH-GHR, PTN-SDC2, PTN-SDC4, and DLK1-NOTCH3, were relatively active in the pituitary–kidney axis. These ligand–receptor pairs mainly target proximal tubule cells, principal cells, the loop of Henle, intercalated cells, pericytes, mesangial cells, and fibroblasts, and these cells are related to physiological processes, such as substance reabsorption, angiogenesis, and tissue repair. Our results suggested that the pituitary gland might directly regulate kidney function by secreting multiple hormones or cytokines and indicated that the above ligand–receptor pairs might represent a new research focus for studies on kidney function or kidney disease.

## Introduction

All organs in the human body are connected directly or indirectly and work in conjunction to maintain the homeostasis of the internal environment under physiological or pathophysiological conditions. The brain and kidney are vital organs of the human body, and novel findings on brain-kidney crosstalk have been reported in recent years^1–3^. Under normal conditions, the brain is involved in kidney functions mainly through the neuroendocrine and autonomic nervous system. The pituitary gland produces cytokines, such as arginine vasopressin (AVP), which act on distal tubules or collecting ducts to regulate water reabsorption. Although the pituitary gland can produce various hormones targeted to specific organs, few of these hormones have been investigated to determine their effect on the kidney. Many kidney diseases are directly or indirectly related to hormone secretion dysfunction because hormones released from the pituitary gland can regulate a series of physiological processes, such as metabolism, cell behavior, tissue repair, which are relevant to kidney functions^4–6^. Thus, the effect of these hormones on kidney components must be determined.

Research has found that cerebrovascular disease, uremic encephalopathy, and cognitive malfunction are prevalent in patients with acute kidney injury or chronic kidney disease^7–10^. Toxic metabolite retention, high proinflammatory cytokine levels, and hyperosmolar status could lead to brain damage in patients with kidney disease. Thus, the brain and kidney could influence each other in a two-way feedback manner. As a main neuroendocrine component of the brain, the pituitary gland is an important component of the feedback loop of the internal environment and is responsible for producing hormones or cytokines that regulate target organ function. Moreover, hormones or cytokines produced by different cell types in the pituitary provide the basis for communication between the pituitary and target organs.

In our current study, single-cell RNA sequencing data on the pituitary and kidney of C57BL/6 mice were used to reveal the cell communication between the pituitary and kidney cell types. The findings may provide a new perspective for studying kidney function regulation or identifying new targets for kidney disease.

## Results

### Combination of pituitary and kidney single-cell transcriptomic data

**Figure 1:**
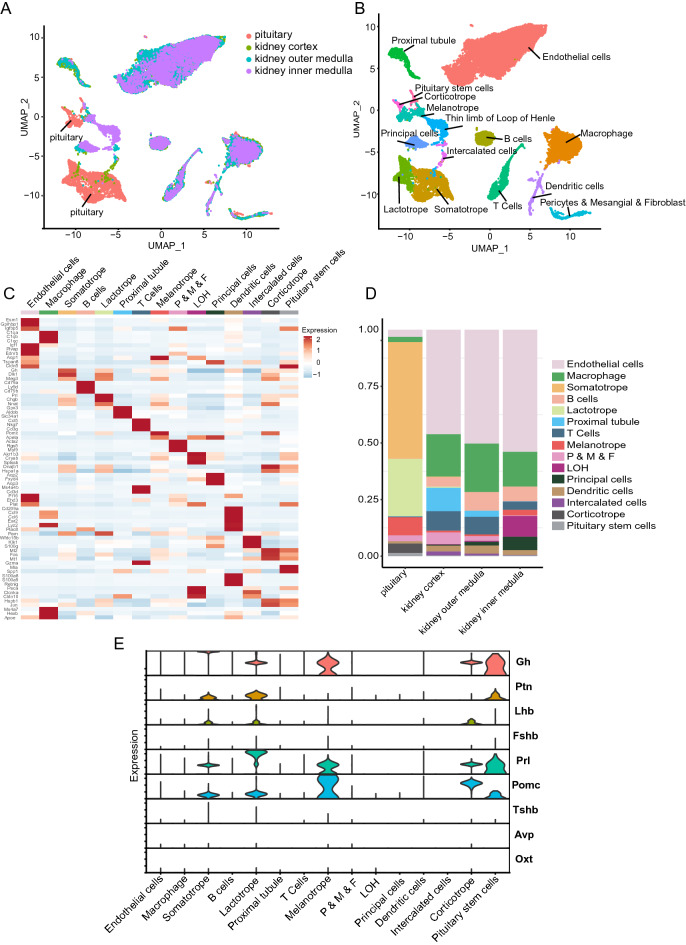
Combination of the pituitary and kidney single-cell transcriptomic data. (**A**) UMAP of the sample distribution in cell clusters based on a combination of pituitary and kidney single-cell transcriptomic data. (**B**) Cell cluster annotations of the combined pituitary and kidney single-cell transcriptomic data. (**C**) Heatmap of top three highest expressed genes of each cell type. (**D**) Bar plot of the percentage of cell types in each sample. (**E**) Expression of classical hormones of the pituitary.

Pituitary and kidney single-cell RNA sequencing data for C57BL/6 mice were obtained from the GEO database (GSE120410 and GSE129798). The two datasets were combined for analysis, and the R package “Harmony” was used to remove batch effects. After stringent raw data processing and filtration, 27815 cells in total were identified, among which, 22961 kidney cells and 4854 pituitary cells were used for further analysis. Cell clustering showed that pituitary cell clusters were clearly separated from kidney cell clusters, which indicated that the quality of the data used for analysis is good (Fig. 1A and Supplemental Fig. 1). Based on classical cell markers of pituitary and kidney cells, cell cluster annotation showed that most kidney and pituitary cell types were obtained (Fig. 1B). The top 3 genes in each cell cluster in terms of expression were identified, and each cell type could be distinctly distinguished (Fig. 1C). The proportion of each cell type varied among the different samples (Fig. 1D). Classical hormone expression in the pituitary showed that growth hormone (GH), pleiotrophin (PTN), luteinizing hormone, prolactin, and proopiomelanocortin were expressed by the pituitary cells. However, the expression of follicle-stimulating hormone, thyroid stimulating hormone, AVP, and oxytocin was not observed (Fig. 1E), which may have been caused by technical reasons, such as cell loss during single-cell preparation or failure to capture these cell types because the pituitary gland of mouse is tiny and difficult to handle.

### Exploration of pituitary and kidney cell–cell interaction patterns

**Figure 2:**
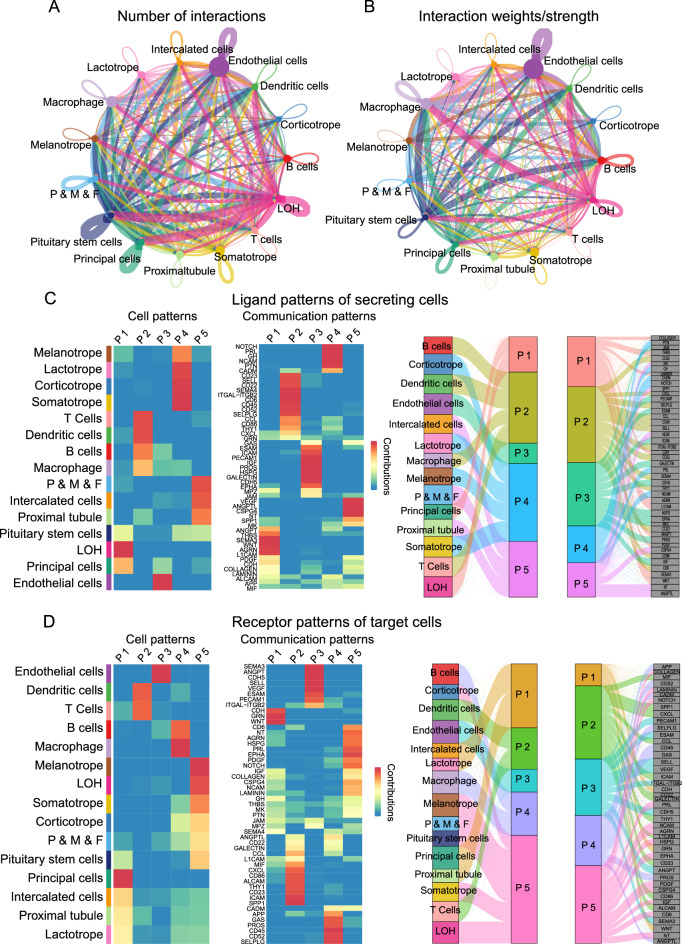
Exploration of the pituitary and kidney cell–cell interaction patterns. (**A**) Circle plot of the ligand–receptor pairs of all cell types, with the thickness of the string representing the number of ligand–receptor pairs. (**B**) Circle plot of the ligand–receptor pairs of all cell types, with the thickness of the string representing the weight of the ligand–receptor pairs. (**C**) Ligand patterns of each cell type, with each pattern representing a set of ligands that have similar properties. The color range represents the contribution scores of patterns or ligands (left panel), and the river plot shows the contribution of ligands to different patterns and the contribution of patterns to different cell types (right panel). (**D**) Receptor patterns of each cell type, with each pattern representing a set of receptors that have similar properties. The color range represents the contribution scores of the pattern or receptor (left panel), and the river plot shows the receptors that contribute to different patterns and the patterns that contribute to different cell types (right panel).

Cell–cell interactions of these cell types were explored based on the ligand–receptor expression of the cells. The ligand–receptor database CellChatDB was used to match all ligand–receptor pairs for each cell type. The numbers and weights of the ligand–receptor pairs were calculated and described, and the loop of Henle, endothelial cells, pituitary stem cells, pericytes, mesangial cells, fibroblasts, and somatotropes were relatively active among all cell types (Fig. 2A,B). We also calculated the cell communication patterns among the cell types based on the similarity of ligand and receptor expression among the different cell types. We found that the ligand patterns of secreting cells were clustered automatically based on similar cell types; patterns 1, 3, and 5 mainly included clusters of kidney cells, and adhesion molecules like L1CAM and ICAM, growth factors like PDGF, VEGF, and IGF were included in these patterns; pattern 2 mainly included clusters of immune cells, and chemokines like CXCL and CCL, Cluster of Differentiation of immune cells like CD45, CD52, and CD86 were included in pattern 2; and pattern 4 mainly included clusters of pituitary cells, hormones like PTN, GH, and PRL were included in pattern 4. The ligands for each pattern are shown in Fig. 2C. Receptor patterns were also explored, and the results showed that most of the immune and kidney cell types were active among the five receptor patterns. The receptors of each pattern are shown in Fig. 2D.

### Most contributing ligand–receptor pairs between pituitary cell and other cell types

**Figure 3:**
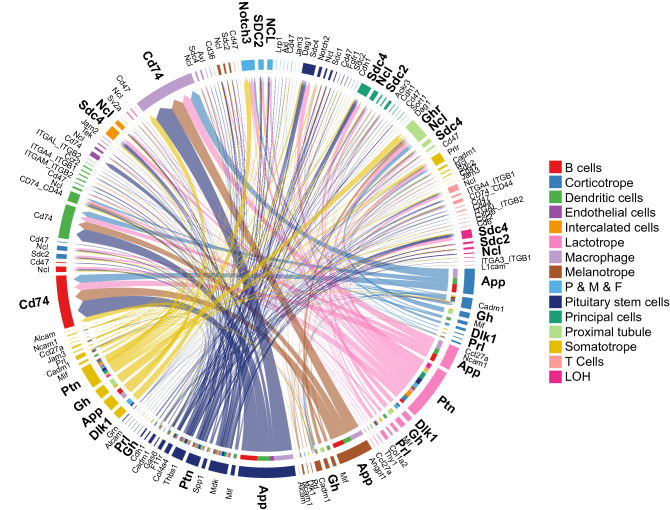
Cord plot of ligand–receptor pairs among the pituitary cell types. Ligand–receptor pairs in somatotropes, lactotropes, melanotropes, corticotropes and pituitary stem cells are shown, and highly active ligand–receptor pairs are highlighted.

To investigate the major ligand–receptor pairs between pituitary cells and other cell types, all ligand receptor pairs of somatotropes, lactotropes, melanotropes, corticotropes, and pituitary stem cells were observed (Fig. 3 and Supplemental Fig. 2). We found that several ligand–receptor pairs, such as GH-GHR, PTN-SDC2, PTN-SDC4, PTN-NCL, APP-CD74, and DLK1-NOTCH3, have higher weights between pituitary cells and other cell types. Among these pairs, APP-CD74 was mainly observed between resident cells and immune cells. Functional and structural analyses were carried out for all ligand–receptor pairs (Supplemental Fig. 3).

### GH, PTN, and DLK1 signaling network among all cell types

**Figure 4:**
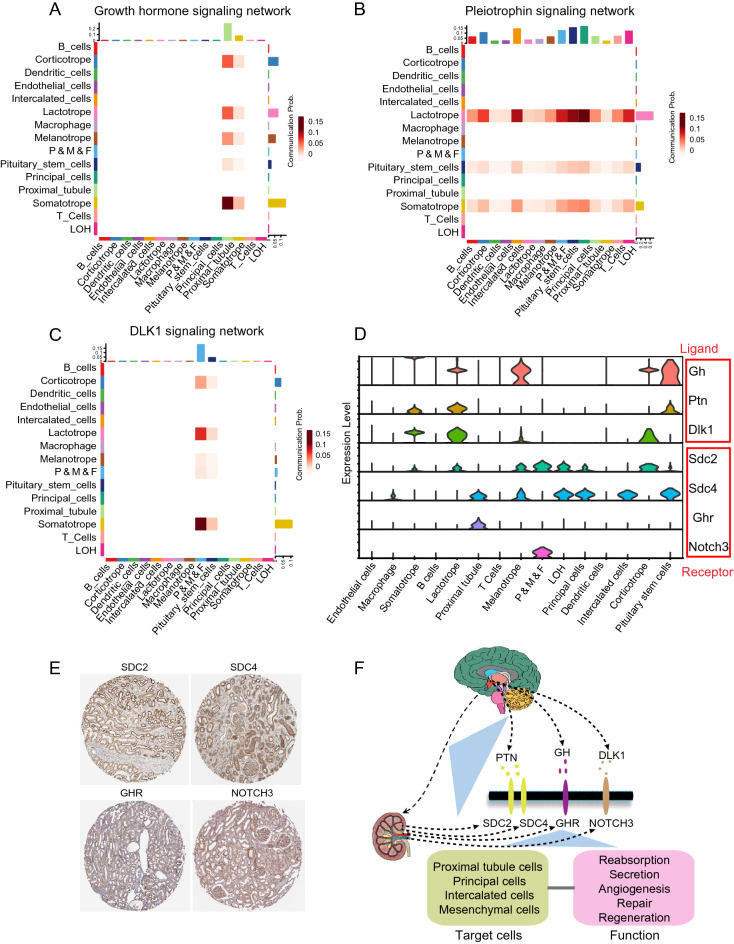
GH, PTN, and DLK1 signaling network among all cell types. (**A**) Communication probability of the GH ligand–receptor pair was calculated for cell–cell communication, with the color range representing the communication probability. (**B**) Communication probability of the PTN ligand–receptor pair was calculated for cell–cell communication, with the color range representing the communication probability. (**C**) Communication probability of the DLK1 ligand–receptor pair was calculated for cell–cell communication, with the color range representing the communication probability. (**D**) Expression of most contribution ligand–receptor pairs among the cell types. (**E**) Immunohistochemical staining of GHR, SDC2, SDC4, and NOTCH3 in the human kidney. (**F**) Hypothesis of the pituitary–kidney axis.

To investigate the specificity of the GH, PTN, and DLK1 signaling network of pituitary cells, we calculated the communication probability between each cell-type pair. The GH signaling network was most active between the proximal tubule and pituitary cells (Fig. 4A). The PTN signaling network was most active among pericytes, mesangial cells and fibroblasts, the loop of Henle, proximal tubule, principal cells, intercalated cells, somatotropes, lactotropes, and pituitary stem cells (Fig. 4B). The DLK1 signaling network was most active among pericytes, mesangial cells, fibroblasts, and pituitary cells (Fig. 4C). GH was expressed in all pituitary cell types and showed relatively higher expression in somatotropes; PTN was mainly expressed in somatotropes, lactotropes, and pituitary stem cells; and DLK1 was mainly expressed in somatotropes, lactotropes, and corticotropes (Fig. 4D). SDC2 was mainly expressed in pericytes, mesangial cells, fibroblasts, and the loop of Henle; SDC4 was mainly expressed in proximal tubules, the loop of Henle, principal cells, and intercalated cells; GHR was only expressed in proximal tubules; and NOTCH3 was only expressed in pericytes, mesangial cells, and fibroblasts (Fig. 4D). To validate the expression of these receptors in the kidney, immunohistochemical staining results were searched in the Human Protein Atlas, and we found that SDC2, SDC4, GHR, and NOTCH3 are highly expressed in human kidneys, which is consistent with our data analysis.

## Discussion

Organs of mammals are separated but united, and they fulfill different biological functions and exhibit coordination to maintain homeostasis in the body. Thus, good communication between organs is extremely important. The brain represents the “commander” of the body and is often involved in regulating organ function through neuroregulation or neuroendocrine processes. Recently, researchers showed that brain-kidney crosstalk plays a role in normal kidney functions or abnormal kidney conditions^1–3^. AVP is a well-known hormone produced by the pituitary that targets the kidney to regulate the reabsorption of water. However, whether other hormones produced by the pituitary also have an effect on kidney functions has not been clarified. Therefore, we used single-cell RNA sequencing data for the pituitary and kidney to explore the hormones or cytokines that act on the kidney and identify the potential underlying mechanisms.

After removing batch effects, high-quality merged pituitary and kidney single-cell transcriptomics data were obtained, and the results showed a distinct separation between pituitary and kidney cell clusters (Fig. 1A and Supplemental Fig. 1). We could see some cells cluster of pituitary and kidney clustered together which mainly are some resident macrophage or dendritic cells in Fig. 1A. Cell annotation showed that most of the pituitary and kidney cell types were captured. Thus, the combined data on the pituitary and kidney used for analysis were of high-quality and reliable. However, the expression of certain classical hormones, such as Fshb, Tshb, Avp, and Oxt (Fig. 1E), was not observed in pituitary cells, which may have been related to the difficulty of preparing single-cell suspensions of pituitary cells, owing to the relatively tiny pituitary of mice, resulting in tissue or cell loss during the preparation of single-cell suspensions. Further, the number of kidney cells was much higher than that of pituitary cells in the current data, and because of the size and properties of the pituitary, certain rare cell types may not have been captured during sample preparation.

Based on the amounts and expression levels of the ligand–receptor pairs among different cell types, we inferred the communication patterns of secreting and targeting cells. As shown, the ligand–receptors patterns were automatically clustered among similar cell types, such as kidney resident cells, immune cells, and pituitary cells, which also indicates that the current data used for analysis are of good quality (Fig. 2C,D). In addition, these ligand or receptor patterns could be used as featured modules to investigate pituitary–kidney communication in future studies.

All ligands of pituitary cells were then studied. Ligand–receptor pairs, such as GH-GHR, PTN-SDC2, PTN-SDC4, PTN-NCL, APP-CD74, and DLK1-NOTCH3, were highly active in the pituitary cell types (Fig. 3). Growth hormone is related to organ development, cell reproduction, cell regeneration, etc.; however, it also has other functions related to metabolism, such as increasing calcium retention, promoting lipolysis, and promoting gluconeogenesis. Therefore, growth hormone could target many cell types to regulate different biological process. In the kidney, GH is related to renal development, glomerular functions, and tubular handling of sodium, calcium, phosphate, and glucose^16^. GHR is expressed in kidney cells, with the most abundant expression observed in proximal tubules^17,18^. Our data support the above findings from a single-cell perspective, and the highest GHR expression was primarily observed in proximal tubules, which primarily perform the substance reabsorption function. These findings indicate that the pituitary could directly regulate kidney reabsorption by secreting GH, and it may further coordinate via AVP to maintain the electrolyte concentration and osmolality of blood. Moreover, the pituitary may also be involved in regenerating tubular cells.

Although studies have shown that PTN is associated with the development of the nervous system, few studies have focused on the effect of PTN on the kidney. However, our data showed that PTN receptors, such as SDC2 and SDC4, are expressed in several cell types in the kidney. Few research studies have focused on SDC2 in the kidney; thus, this might be a novel study direction for kidney cell regeneration. SDC4 is related to tubulointerstitial fibrosis, and interference in SDC4 could relieve tubulointerstitial fibrosis in mouse models of unilateral ureteric obstruction and aristolochic acid nephropathy^19^. These findings indicated that PTN-SDC4 may be involved in kidney repair and regeneration; moreover, overactivation of the PTN-SDC4 signaling pathway may cause abnormal repair functions. Thus, our data imply that PTN-SDC2 or PTN-SDC4 may play a role in the pituitary–kidney axis.

DLK1 is a member of the EGF-like family of homeotic proteins, and it has been shown to inhibit the activity of the NOTCH family, which is involved in adipogenesis and the differentiation of preadipocytes into adipocytes^20^. Although DLK1 is not a classical hormone secreted by the pituitary, our data revealed relatively high DLK1 expression in somatotropes, lactotropes, and corticotropes (Fig. 4D). Therefore, DLK1 secreted by the pituitary may be involved in adipogenesis of the whole body. However, whether DLK1 could regulate other biological processes in addition to adipogenesis has not been clarified. Our current data showed that NOTCH3 expression was relatively high in the mesenchymal cells of the kidney. Pericytes, mesangial cells, and fibroblasts have similar embryonic progenitors and also share some common cell markers, such as PDGFRβ and αSMA. These cell types present strong proliferation and differentiation and are often involved in local tissue repair or angiogenesis. This function is similar to that of DLK1, which influences the differentiation of preadipocytes. Thus, the DLK1-NOTCH3 pair may influence the proliferation and differentiation of mesenchymal cells in the kidney.

Cell–cell interactions between the pituitary and kidney were clearly revealed in the current study based on a combined analysis of single-cell RNA sequencing data. Ligand–receptor pairs found among all cell types of the pituitary and kidney were described, and more direct connections between the pituitary and kidney were revealed. GH-GHR, PTN-SDC2, PTN-SDC4, and DLK1-NOTCH3 may play important roles in the pituitary–kidney axis, which may be related to the target cells and their functions (Fig. 4F). We provide fundamental evidence that the pituitary might regulates various kidney functions through multiple signaling pathways. A limitation of the current study is that the data were not obtained from one study. Moreover, although we applied the batch effect correction method to reduce the impact of confounding factors, bias was inevitable and may have influenced the data analysis. Moreover, the mechanism associated with our findings should be validated in the future.

## Methods

### Data sources

Single-cell RNA sequencing data for the pituitary (n = 6) and kidney (n = 3) of C57BL/6 mice from 10 × Genomics were used for the analysis. Data were collected from the Gene Expression Omnibus (GEO) database (GSE120410, GSE129798). All relevant analyses were carried out in R (version 4.1.3).

### Single-cell raw data quality control

The Seurat R package (version 4.1.0) was applied for further analyses^11^, with the “MergeSeurat” function used to merge multiple datasets. According to the median number of genes and the percentage of mitochondrial genes in the kidney samples, cells with < 200 and > 2,500 genes, a mitochondrial gene percentage of > 10%, and a hemoglobin gene percentage > 3% were filtered. Gene expression matrices were normalized to the total cellular unique molecular identifiers (UMIs) count. The normalized expression was scaled by regressing the total cellular UMI counts. After data normalization, all highly variable genes in single cells were identified after controlling for the relationship between average expression and dispersion. Subsequently, we performed a principal component analysis with variable genes as the input and identified significant principal components (PCs) based on the “jackstraw” function. Twenty PCs were selected as the input for uniform manifold approximation and projection.

### Batch effect correction and cell type identification

Since these data were obtained from different samples, the R package “Harmony” was used for batch correction (version 1.0) to prevent the batch effect from disrupting downstream analyses ^12^. At a resolution of 0.8, cells were clustered using the “FindClusters” function and classified into 28 different cell types. Next, we used the “FindAllMarkers” function to identify differentially expressed genes of each cell type. Cluster identification was performed based on the top differentially expressed genes of each cluster, and each gene was manually checked on the CellMarker database or in published articles to match the cell types.

### Cell–cell interaction analysis

To evaluate cell–cell interactions, the R package CellChat (version 1.1.3) was used to evaluate the expression of pairs of ligands and receptors within cell populations based on the databases CellChatDB and STRINGDB^13,14^. The function “computeCommunProb” was used to calculate the communication probability and infer the cellular communication network. The communication probability of a signaling pathway was computed by summarizing the probabilities of its associated ligand–receptor pairs and the communication probability here only represents the interaction strength and is not exactly a probability. The function “computeCommunProbPathway” was used to infer the cell–cell communication at a signaling pathway level, and the function “aggregateNet” was used to calculate the aggregated cell–cell communication network. The expression of key receptors in the human kidney were validated using the Human Protein Atlas^15^.

## Supplementary Information

Supplementary Information.

## Data Availability

The datasets analysed during the current study are available in the GEO repository, GSE120410 and GSE129798.
