# Supplementary material for: Single-cell RNA sequencing data analysis suggests the cell–cell interaction patterns of the pituitary–kidney axis
Source: Sci Rep. 2022 Jul 1;12:11147. doi: 10.1038/s41598-022-14680-2 (PMC9249760; doi:10.1038/s41598-022-14680-2)
Supplement: Supplementary file 1 — Supplementary Information. [file 41598_2022_14680_MOESM1_ESM.pdf]

Single-cell RNA sequencing data analysis suggests the cell-cell interaction patterns of the pituitary-kidney axis

Yiyao Deng[1], Jingjing Da[1, 2, 3], Jiali Yu[1, 2, 3], Chaomin Zhou[1], Jing Yuan[1,*], Yan Zha[1, 2, 3,*]

[1]Department of Nephrology, Guizhou Provincial People's Hospital, 83, Zhongshan Road, Nanming District, Guiyang, 550002, Guizhou, China.

[2]School of Medicine, Guizhou University, Guiyang, 550025, Guizhou, China.

[3]NHC Key Laboratory of Pulmonary Immunological Disease, Guizhou Provincial People's Hospital, Guiyang, 550002, Guizhou, China.

Running title: The cell-cell interaction patterns of the pituitary-kidney axis

*Corresponding author: zhayan72@126.com, yuanjinger@126.com.

Tel: +86 13639106123

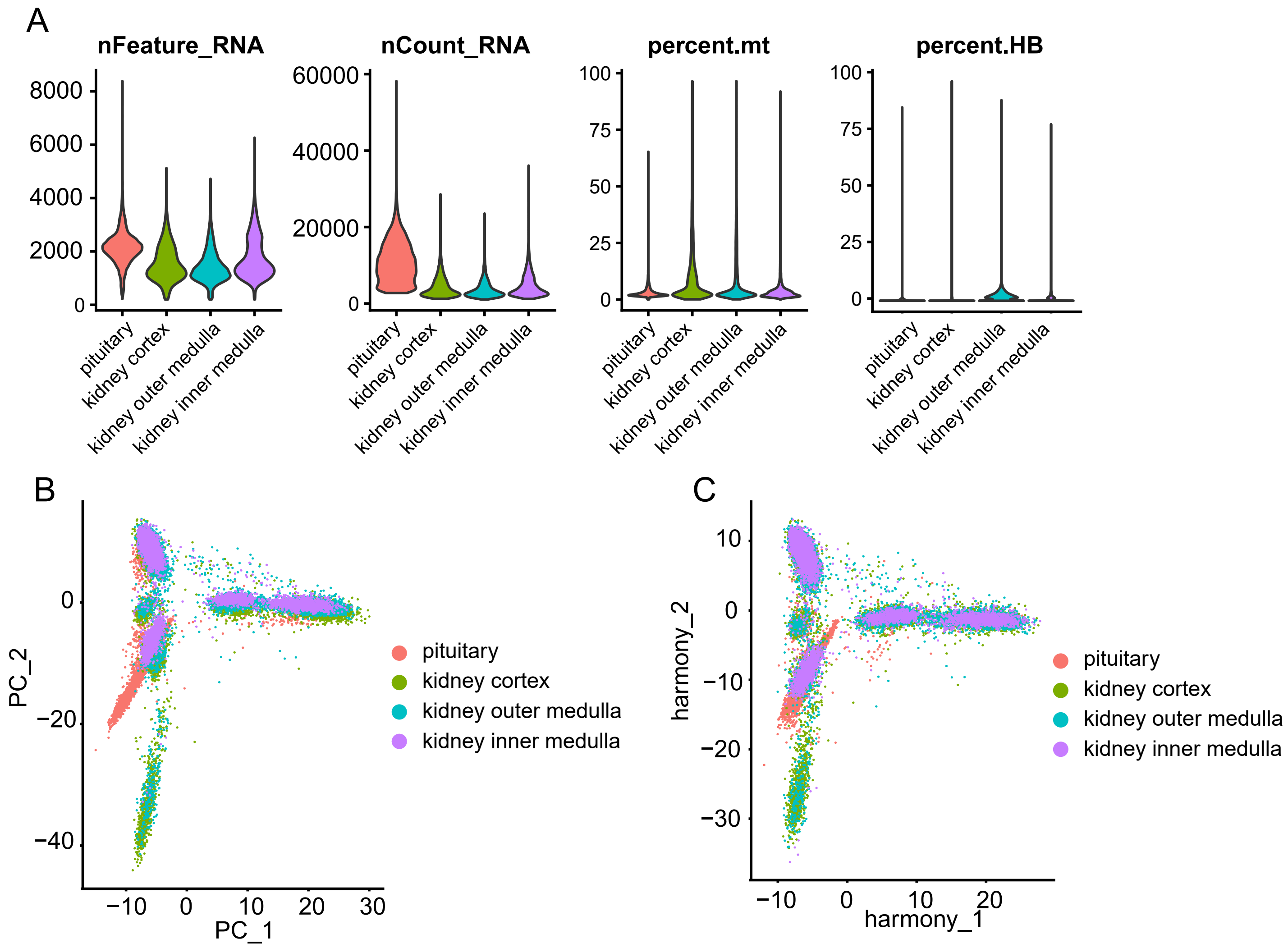

Supplemental Figure 1. Quality control of the combined pituitary and kidney single-cell sequencing data. (A) Genes, unique molecular identifier (UMI) counts, mitochondrial gene percentage, and hemoglobin gene percentage of the combined data. (B) Cell distribution in PC1 and PC2 before batch effect correction. (C) Cell distribution in PC1 and PC2 after batch effect correction by using "Harmony".

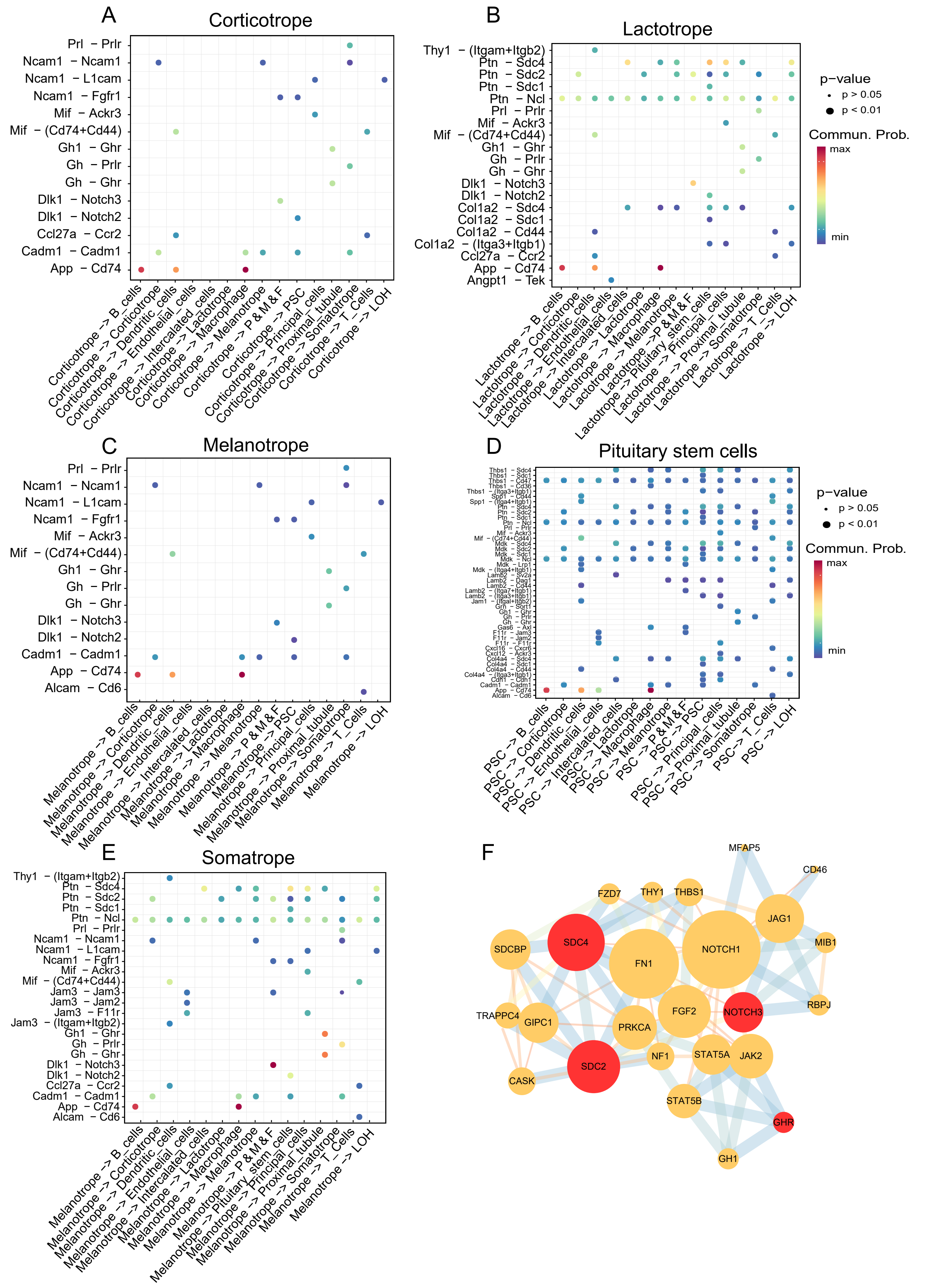

Supplemental Figure 2. Ligand-receptor pair communication probability among the pituitary cell types. Ligand-receptor pair communication probability for (A) corticotropes, (B) lactotropes, (C) melanotropes, (D) pituitary stem cells, and (E) somatotropes. (F) Protein-protein interaction network of GHR, SDC2, SDC4, and NOTCH3.

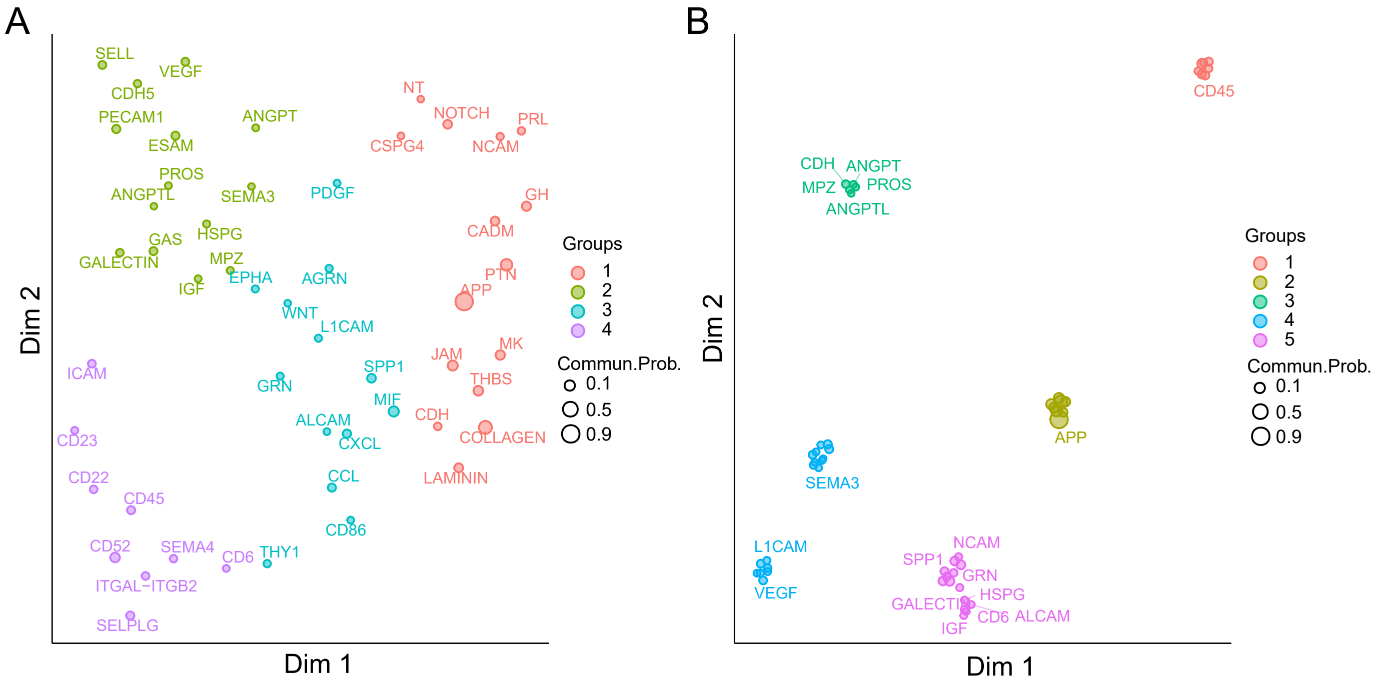

Supplemental Figure 3. Functional and structural similarity of ligand-receptor pairs. (A) Functional of ligand-receptor pairs. (B) Structural similarity of ligand-receptor pairs.
